# Effective photoacoustic absorption spectrum for collagen-based tissue imaging

**DOI:** 10.1117/1.JBO.25.5.056002

**Published:** 2020-05-13

**Authors:** Eunwoo Park, Yong-Jae Lee, Changho Lee, Tae Joong Eom

**Affiliations:** a Gwangju Institute of Science and Technology, Advanced Photonics Research Institute, Gwangju, Republic of Korea; b Chonnam National University, Medical School and Hwasun Hospital, Department of Nuclear Medicine, Hwasun, Republic of Korea

**Keywords:** photoacoustic imaging, short-wave infrared, absorption spectrum, collagen, fiber laser

## Abstract

**Significance:** Collagen is a basic component of many tissues such as tendons, muscles, and skin, and its imaging helps diagnose and monitor treatments in a variety of fields, including orthopedics. However, due to the overlapping peaks of the absorption spectrum with water in the short-wave infrared region (SWIR), it is difficult to select an optimal wavelength and obtain the photoacoustic (PA) image for collagen-based tissues. Therefore, an additional approach to selecting the proper wavelength is needed.

**Aim:** The aim of this study is to derive an effective PA absorption spectrum of collagen to select the optimal wavelength for high-sensitive PA imaging (PAI).

**Approach:** We measure the absorption spectrum by acquiring the PA signal from various collagen-based samples. To derive an effective PA absorption spectrum in the SWIR band, the following two parameters should be considered: (1) the laser excitation for generating the PA signal and (2) the absorption spectrum for water in the SWIR band. This molecular intrinsic property suggests the optimal wavelength for high-sensitive PAI of collagen-based samples.

**Results**: PA absorption spectral peaks of collagen were found at wavelengths of 1200, 1550, and 1700 nm. Thereby, the PA signal increased by up to five times compared with the wavelength commonly used in collagen PAI. We applied a pulsed fiber laser with a center wavelength of 1560 nm, and the three-dimensional PA image of a collagen patch was obtained.

**Conclusions:** The effective PA absorption spectrum contributes to the improvement of the PA image sensitivity by presenting the optimal wavelength of the target samples.

## 1 Introduction

Photoacoustic imaging (PAI) is a promising hybrid imaging technique for biomedical applications, combining optical contrast and acoustic resolution.^1^ PAI provides anatomical and functional information based on strong optical absorption sensitivity.^1^^–^^3^ The optimization of the wavelength according to the target molecule can enhance the selectivity of the visualization of target organs and allows for the extension into functional PAI such as multispectral imaging.^4^^,^^5^ Typically, in the ultraviolet region (10 to 400 nm), the imaging of cell nuclei is performed utilizing the strong absorption by DNA and RNA at wavelengths of ∼260  nm.^6^ In the visible (VIS) region (400 to 700 nm), vascular imaging is typically performed using wavelengths of around 532 nm.^7^ In the first window of the near-infrared region (NIR-I, 700 to 1000 nm), previous research has achieved relatively deep vascular imaging by taking advantage of the low light attenuation.^8^ Additionally, to delineate transparent organs such as lymph nodes, gastrointestinal tracts, and bladders, labeled imaging has also been performed using several contrast agents, such as indocyanine green or methylene blue.^9^^–^^12^ The second window of the NIR (NIR-II) region, also called short-wave infrared region (SWIR, defined here as 1100 to 2000 nm), is of current interest due to the relative low levels of scattering and the high-maximum permissible exposure compared with the NIR-I region.^12^^–^^15^ Additionally, in the SWIR band, we find the optical absorption peaks of water, collagen, and lipids, three major molecules of human tissues.^15^^–^^17^

Collagen is an important substance that is the basis of tissues such as the tendons, ligaments, and muscles in our body, and its imaging helps with the diagnosis and monitoring of treatments in a variety of fields, including orthopedics, dermatology, and cardiology. Recently, the imaging of collagen and collagen-based tissues using the PAI method was reported.^18^^,^^19^ Even though the optical absorption by collagen is relatively high in the VIS and NIR-I wavelength ranges, it still requires precise optical alignment and a complex acoustic capturing process to measure the PA signals of collagen in biological tissue due to the dominant concentration of oxy-/deoxy-hemoglobin (Hb) in whole blood and its morphological proximity to the blood vessels. Therefore, one solution involves looking for an effective collagen absorption spectrum in the SWIR band, which presents two main benefits: (1) avoiding the interference from whole blood and (2) achieving almost an order of magnitude higher absorption by collagen than with the NIR-I band.^15^^–^^17^ The absorption peaks of collagen in the SWIR band were reported at wavelengths of 1200, 1500, and 1725 nm.^17^^,^^20^^,^^21^ Unfortunately, these peaks overlap with the absorption peaks of water, which are found at wavelengths of 1180, 1430, and 1930 nm.^17^^,^^22^ This is a critical issue in delivering light into collagen samples. Normal PAI systems utilize water or water gel as the medium for acoustic propagation, so high-absorption losses during laser delivery may lead to insufficient PA signals. To derive an effective PA absorption spectrum for collagen in the SWIR band, the following two parameters should be considered: (1) the laser excitation for generating the PA signal and (2) the absorption spectrum for water in the SWIR band.

In this paper, we measure the absorption spectrum by acquiring the PA signal from various collagen-based samples to find the optimal wavelength for the particular PA application. The effective PA spectrum of collagen can be calculated by measuring the absorption spectrum of PA signals and the absorption spectrum of water in the SWIR band. The calculated collagen PA spectrum was compared with the optical absorption spectra presented in previous research. To demonstrate the utility of the effective PA spectrum, a three-dimensional (3-D) PA image of a collagen sample was obtained using the selected wavelength.

## 2 Materials and Methods

### 2.1 Effective Photoacoustic Absorption Spectrum

According to the PA effect equation, the local pressure rise $p_{0}$ can be expressed as follows:^23^

$$p_{0}(\lambda )=\Gamma \eta_{\mathrm{th}}\mu_{a}(\lambda )\cdot F(\lambda ).$$ (1)

When laser pulses with optical fluence F irradiate the target, the chromophore in the sample absorbs photon energy with absorbance $\mu_{a}$. This is then converted into heat with efficiency $\eta_{\mathrm{th}}$, and the thermal expansion causes a change in the energy of the phonon, which is a vibrational property that depends on the Grüneisen parameter Γ. Since Γ is a function of temperature and $\eta_{\mathrm{th}}$ is a constant of energy conversion efficiency, these are independent variables for wavelength. The effective PA absorption spectrum M, as a function of the wavelength only, can be obtained by dividing the PA signal amplitude A by the laser fluence F:^24^

$$M(\lambda )\equiv \frac{A(\lambda )}{F(\lambda )}.$$ (2)

By normalizing this spectrum, a value proportional to $\Gamma \eta_{\mathrm{th}}\mu_{a}(\lambda )$ can be obtained. This represents the PA effects from the light absorption to the vibration conversion and leads to the information on the components.

To calculate the actual laser fluence reaching the sample, compensation for the optical attenuation from the medium needs to be factored in with the Beer–Lambert law as follows:

$$M(\lambda ,z)\equiv \frac{A(\lambda )}{F_{0}(\lambda )}\cdot \exp(\varepsilon z),$$ (3)

where $F_{0}$ is the laser output fluence after the water tank without the medium and ε is the optical attenuation coefficient of the medium with the path length z (in centimeters). In the case in which water is the medium, the optical attenuation coefficient can be replaced by the optical absorption coefficient. The light scattering coefficient can be ignored because it is eight orders of magnitude lower than the absorption coefficient in the SWIR band.^25^ The entire process for obtaining the effective PA absorption spectrum is summarized as a flowchart in Fig. S1 in the Supplementary Material.

### 2.2 Experimental Setup

**Fig. 1:**
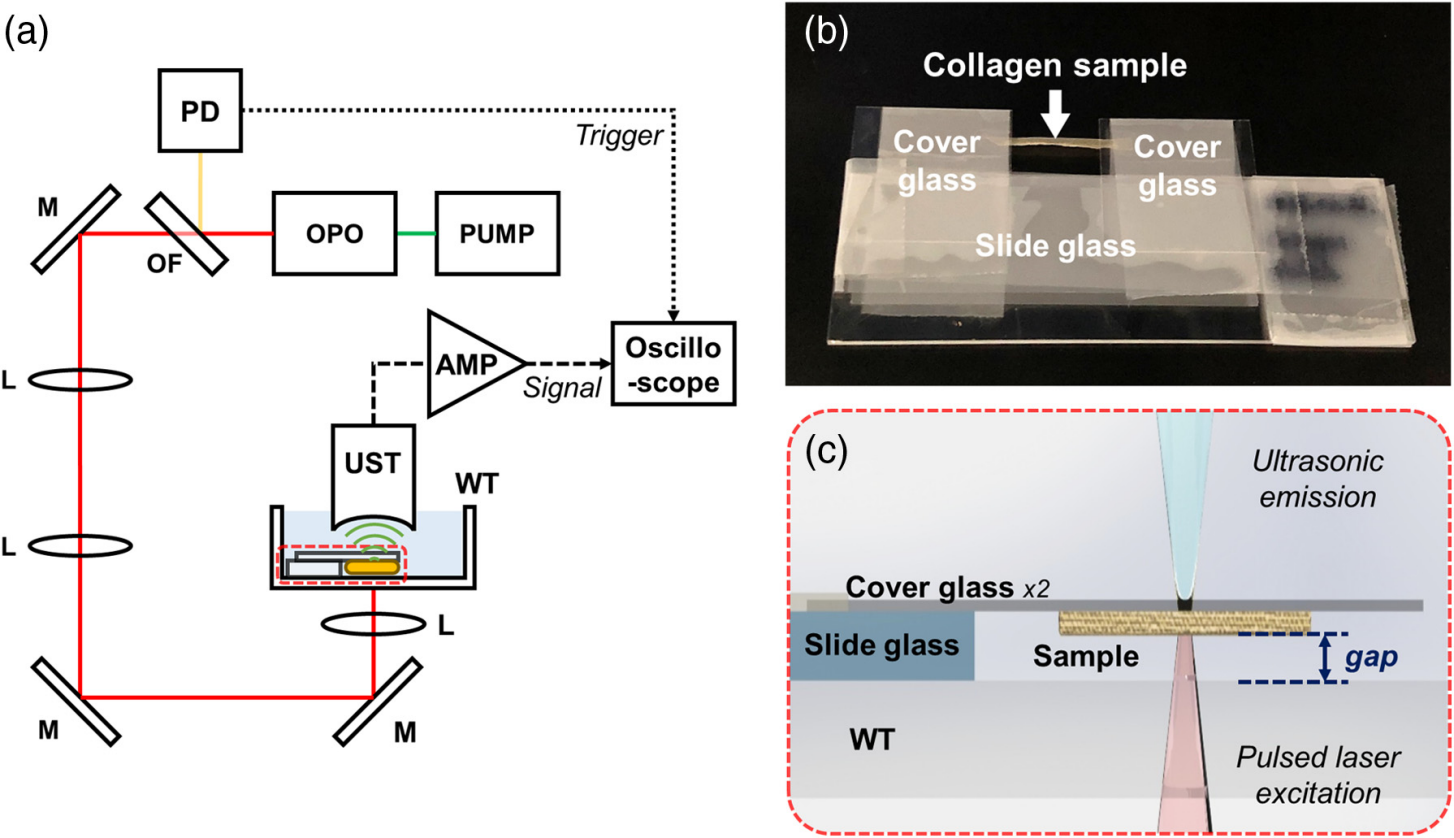
(a) Schematic diagram for the measurement system for the effective PA absorption spectrum. (b) Photograph of the sample preparation. (c) Side view of an enlarged diagram of the sample positioning. OPO, optical parametric oscillator; OF, optical filter; PD, photodetector; M, mirror; L: lens; WT, water tank; UST, ultrasonic transducer; and AMP, amplifier.

Figure 1 shows the schematic of the experimental setup used to measure the effective PA absorption spectrum. The optical parametric oscillator (OPO) (Surelite OPO Plus, Continuum) pulsed laser with a 532-nm wavelength pump (SL EX, Continuum) was used. The laser has a pulse width of 3 to 5 ns, a repetition rate of 10 Hz, and an idler tuning range from 1150 to 1950 nm. The colored glass filter (FGL610, Thorlabs) was used as an optical long-pass filter for the idler output only. An ultrasonic transducer (V324-SM, Olympus NDT) with a center frequency of 25 MHz and a focal length of 0.75 inches, a digital oscilloscope (DS4054, RIGOL), and two amplifiers (ZX60-3018G-S+, 45.2 dB gain, Mini-Circuits) were used for PA signal acquisition.

We used three different samples. The first sample was a collagen patch (CGDerm, CGBio), which is widely used clinically for a bridging graft or reconstruction surgery of collagen-based tissues. The second and third samples were fiber bundles extracted from the tail and Achilles tendons of the mouse (BALB/c Nude, male, 9 to 10 weeks old), respectively. All animal-handling protocols were followed as per the guidelines of the Institutional Animal Care and Use Committee at the Gwangju Institute of Science and Technology, Republic of Korea. Before the experiment, all samples were immersed in deionized water for 1 h to minimize the effect of hydration during the experiment. The samples were hung under two cover glasses as shown in Fig. 1(b). A section on top of the sample was left uncovered by the cover glasses to prevent acoustic signal loss.

The gap between the sample and the water tank, as shown in Fig. 1(c), was controlled to be under 1 mm by stacking on the slide glass. The exact value of the gap for each sample was calculated by the delay time of the acoustic wave for the laser trigger and the sound velocity of the medium. The acoustic delay was recorded by the time difference between two PA signals from the membrane of the water tank and the target sample. The speed of sound in water was assumed to be 1500  m/s, which when multiplied by the time interval gives the path length z used in Eq. (3).

## 3 Results

### 3.1 Measurement of the Effective PA Absorption Spectrum of Collagen

**Fig. 2:**
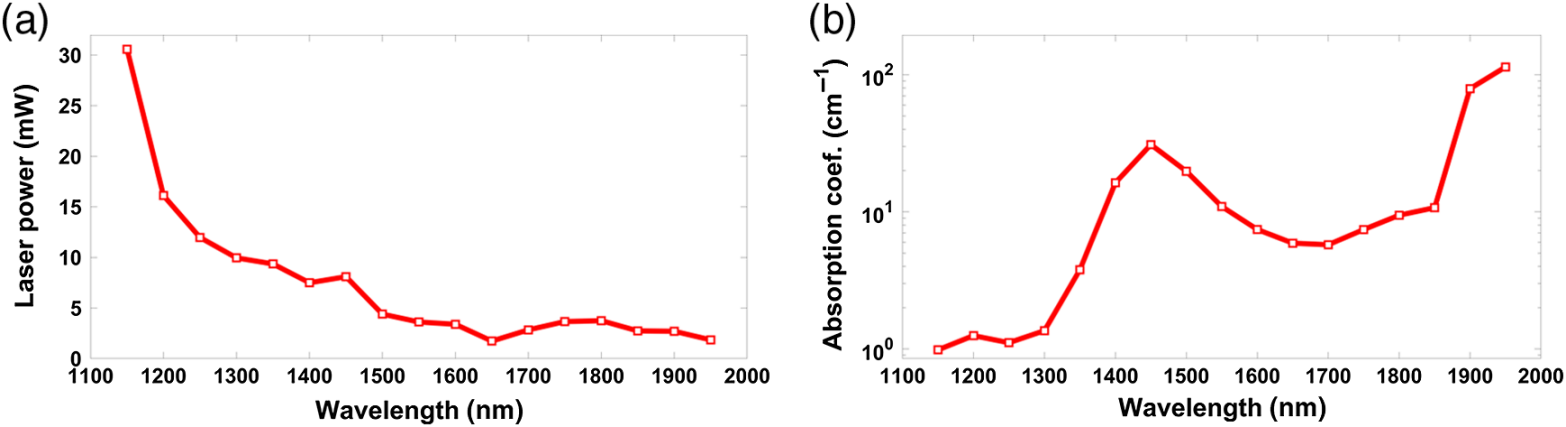
(a) Laser power spectrum and (b) optical absorption spectrum of water.

Before the PA measurement, the optical attenuation of deionized water was measured with an optical power meter (S310, Thorlabs). Using a collimated beam penetrating a cuvette (100-2-20, Hellma Analytics), the laser power with and without water was compared and the optical absorption spectrum of the medium was obtained to calculate each path length. The measurement step size of the laser output wavelength was maintained at 50 nm within the entire tuning range. Figure 2(a) shows the output power of the OPO laser, labeled $F_{0}$, after the unfilled water tank. Figure 2(b) shows the optical absorption coefficient of water, labeled ε.

**Fig. 3:**
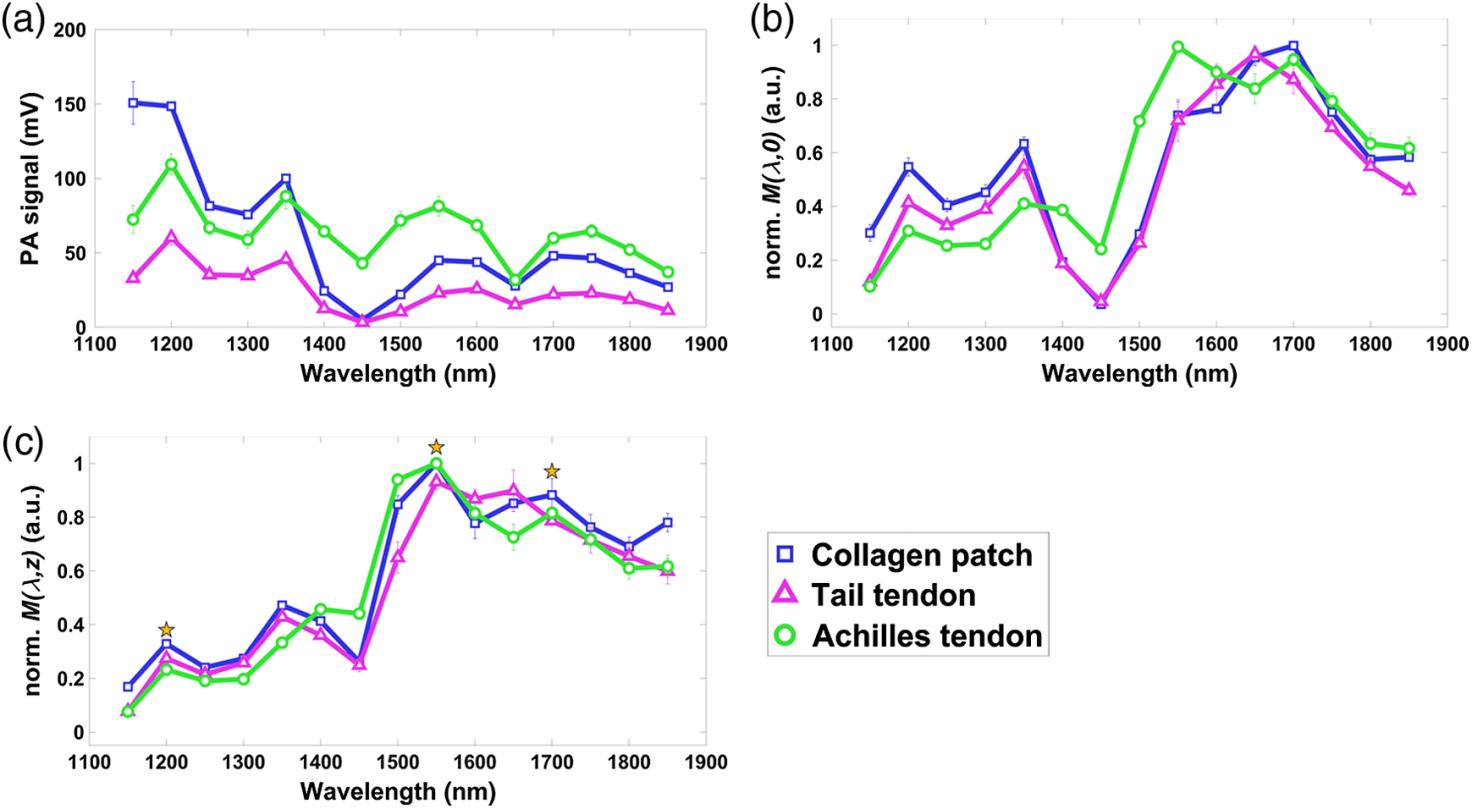
(a) PA signal spectra of collagen-based samples. (b) Normalized effective PA absorption spectra of samples without compensation for optical attenuation. (c) Normalized effective PA absorption spectrum for PAI of collagen with compensation for optical attenuation of water.

The PA signal amplitude spectra of each type of collagen-based tissue are shown in Fig. 3(a). All samples were prepared separately; in total there were two collagen patches, three tail tendon fiber bundles, and four Achilles tendon fiber bundles. By simply dividing the signal amplitude by the laser fluence (after the unfilled water tank), $A(\lambda )/F_{0}(\lambda )$, we arrive at the spectrum shown in Fig. 3(b). The effective PA absorption spectrum of the samples, with compensation for the optical attenuation over the path length z, is shown in Fig. 3(c). Both Figs. 3(b) and 3(c) have been normalized based on the maximum PA values of each sample. Peaks in the effective PA absorption spectrum appear at wavelengths of ∼1200, 1550, and 1700 nm, showing the same spectrum for all three collagen-based tissues.

The averaged peak values were 0.28, 0.98, and 0.85, respectively. In previous research,^15^ normalized absorption values were also measured as 0.29 and 0.96 at wavelengths of 1200 and 1550 nm, respectively. Other measurements of the absorption spectrum of collagen were reported at ∼0.2, 1.3, and 1.5 at wavelengths of 1200, 1500, and 1700 nm, respectively.^16^ The variance in absorption values could be caused by the degree of molecular density.

The strong light attenuation from water at ∼1450  nm makes the PA signal too small to accurately calculate the absorption value. The dry collagen has the optical absorption peak of 1500 nm.^17^^,^^21^ However, the effective PA absorption shifts the peak from 1500 nm to 1550 nm, considering the light absorption of water.

The effective PA absorption spectrum indicates the efficiency with which target molecules can generate PA signals per laser fluence. By compensating for the light attenuation in the medium, this molecular intrinsic property suggests the optimal wavelength for high-sensitive PAI of collagen-based samples (Fig. S1 in the Supplementary Material).

### 3.2 PA Imaging of Collagen-Based Tissue

**Fig. 4:**
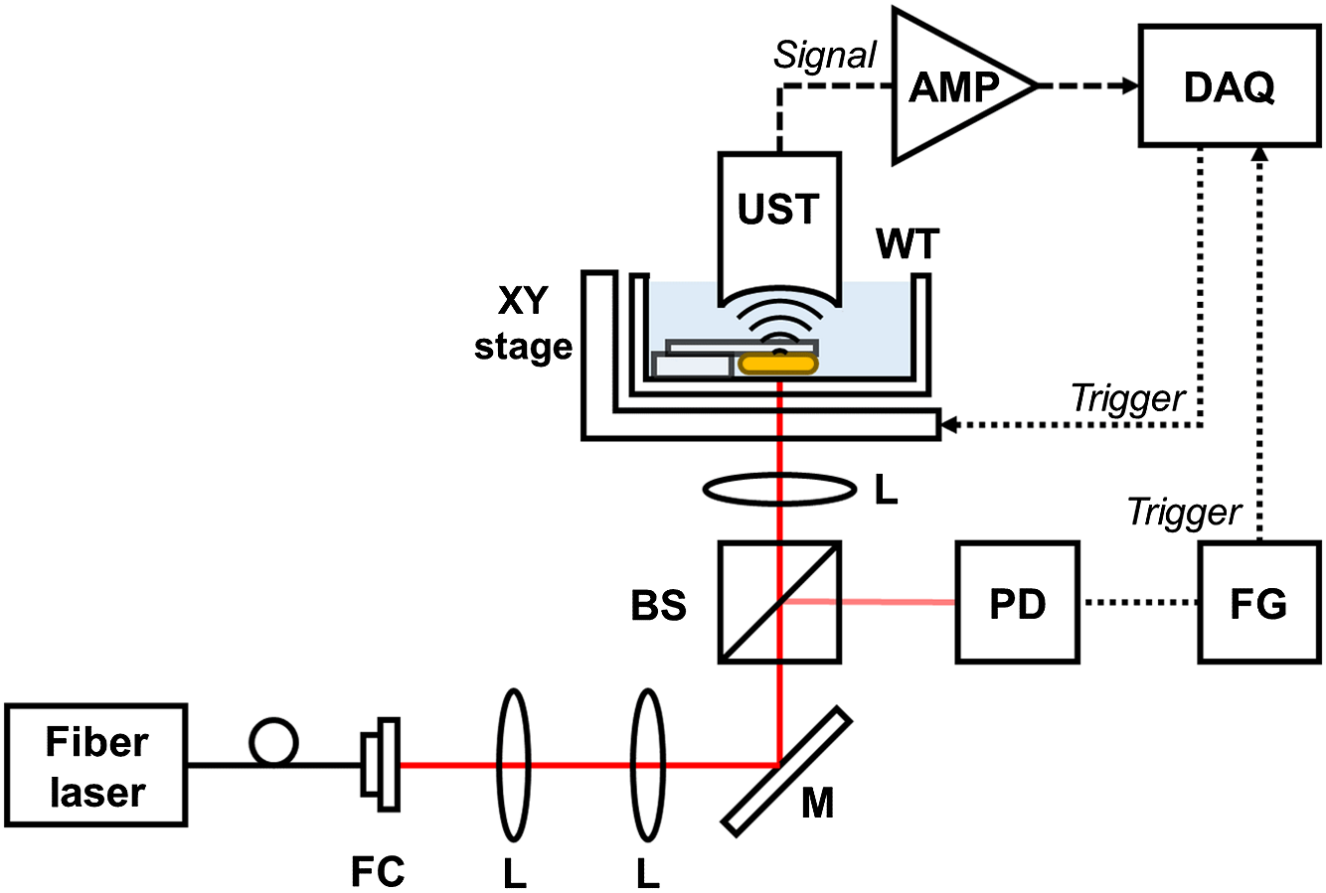
Schematic diagram of the PAI system. FC, fiber coupler; BS, beam splitter; FG, function generator; and DAQ, data acquisition board.

We constructed the PA microscopy (PAM) system for collagen patch sample imaging. Figure 4 shows the schematic diagram of the experimental setup for the PAM system. To exploit the high optical absorption of the collagen sample, a pulsed fiber laser (made in the lab, APRI) was used with a center wavelength of 1560 nm, a pulse width of ˜1ns, and a repetition rate of 5 kHz. A polarizing beam splitter (GL10-C, Thorlabs) was used to split the beam to enter the photodetector (PDA10CF-EC, Thorlabs). The burst signal from a function generator synchronized the laser pulse output, signals for a digitizer (PX14400, Signatec) and an XY-stage (PLS-XY, Thorlabs). The UST and amplifiers for the PA signal measurements are the same as those used previously. The data acquisition software was developed in LabVIEW (National Instruments) with a 1024×2048×512  voxels per volume dataset, and the sampling rate for PA signal acquisition was 400  MS/s.

**Fig. 5:**
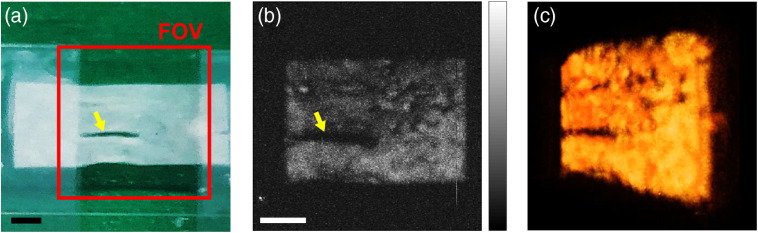
(a) Photograph of a collagen patch sample for PAI. (b) Corresponding PA MAP image. (c) 3-D PA image of the collagen patch (Video 1). FOV, field of view. The inserted scale bar is 1 mm. (Video 1, 5.74 MB, MP4 [URL: https://doi.org/10.1117/1.JBO.25.5.056002.1].)

Figure 5(a) shows a photograph of the collagen patch sample prepared for PAI. The collagen patch was the same sample as measured in the PA spectrum. The red square box indicates the field of view (FOV) of 7  mm×7  mm. The corresponding PA maximum amplitude projection (MAP) image is shown in Fig. 5(b). The PA image of the collagen sample shows the detailed structure of the target patch, including a damaged region marked by the inset yellow arrow. Owing to the sufficient optical absorption at a wavelength of 1560 nm, we obtained a 3-D PA image of the collagen patch as shown in Fig. 5(c). In the MAP image [Fig. 5(b)], the measured signal-to-noise ratio of the collagen patch is ∼53.2  dB (Fig. S2 in the Supplementary Material).

## 4 Discussion and Conclusion

By employing the PA methods, we were able to measure the optical absorption by collagen in the SWIR region and derive its effective PA absorption spectra. The spectra were obtained not only by normalization to the laser fluence but also by compensating for the light loss due to the water medium. The PA absorption peaks of collagen were found at wavelengths of 1200, 1550, and 1700 nm, which are almost identical to the values previously reported in literature.^15^ A wavelength of 1550 nm was chosen as optimal for collagen imaging. We applied a pulsed fiber laser with an output wavelength of 1560 nm and obtained the 3-D PA image of the collagen patch.

Comparing the B-scan images (Fig. S3 in the Supplementary Material) of the collagen patch at wavelengths of 1200, 1310, and 1560 nm with the same laser fluence, the normalized PA signals of the inset box were measured as 0.17, 0.23, and 0.92, respectively. At 1560 nm, that was up to five times over what is possible with wavelengths of 1200 and 1310 nm, which are commonly used in collagen PAI.^18^^,^^19^ This confirms the utility of the effective PA absorption spectrum and optimal wavelength for PAI of collagen-based tissues, thereby contributing to an improvement in the PA image sensitivity.

The accuracy of the spectrum and the sensitivity of the image can be further improved by precisely modeling the PAM system to address issues such as acoustic attenuation and impedance mismatch. This method also allows for the measurement of the effective PA absorption spectrum of many other molecules, such as lipids. Additionally, this method can be extended for high-selective functional PAI in a variety of fields, including orthopedics and cardiology.

## References

[1] Wang L. V. et al., “Photoacoustic tomography: in vivo imaging from organelles to organs,” Science 335(6075), 1458–1462 (2012). SCIEAS 0036-8075 10.1126/science.1216210 22442475 PMC3322413

[r2] Wang X. et al., “Noninvasive laser-induced photoacoustic tomography for structural and functional in vivo imaging of the brain,” Nat. Biotechnol. 21(7), 803–806 (2003). NABIF9 1087-0156 10.1038/nbt839 12808463

[3] Yao J. et al., “High-speed label-free functional photoacoustic microscopy of mouse brain in action,” Nat. Methods 12(5), 407–410 (2015).1548-7091 10.1038/nmeth.3336 25822799 PMC4428901

[4] Yang J. M. et al., “Simultaneous functional photoacoustic and ultrasonic endoscopy of internal organs in vivo,” Nat. Med. 18(8), 1297–1302 (2012).1078-8956 10.1038/nm.2823 22797808 PMC3885361

[5] Taruttis A. et al., “Advanced in real-time multispectral optoacoustic imaging and its applications,” Nat. Photonics 9(4), 219–227 (2015). NPAHBY 1749-4885 10.1038/nphoton.2015.29

[6] Yao D. K. et al., “Optimal ultraviolet wavelength for in vivo photoacoustic imaging of cell nuclei,” J. Biomed. Opt. 17(5), 056004 (2012). JBOPFO 1083-3668 10.1117/1.JBO.17.5.056004 22612127 PMC3602808

[7] Yao J. et al., “Label-free oxygen-metabolic photoacoustic microscopy in vivo,” J. Biomed. Opt. 16(7), 076003 (2011). JBOPFO 1083-3668 10.1117/1.3594786 21806264 PMC3144973

[8] Hai P. et al., “Near-infrared optical resolution photoacoustic microscopy,” Opt. Lett. 39(17), 5192–5195 (2014). OPLEDP 0146-9592 10.1364/OL.39.005192 25166107 PMC4161671

[9] Lee Y. J. et al., “Photoacoustic imaging probe for detecting lymph nodes and spreading of cancer at various depths,” J. Biomed. Opt. 22(9), 091513 (2017). JBOPFO 1083-3668 10.1117/1.JBO.22.9.091513 28444151

[r10] Erpelding T. N. et al., “Sentinel lymph nodes in the rat: noninvasive photoacoustic and US imaging with a clinical US system,” Radiology 256(1), 102–110 (2010). RADLAX 0033-8419 10.1148/radiol.10091772 20574088 PMC2897692

[r11] Yoo S. W. et al., “Biodegradable contrast agents for photoacoustic imaging,” Appl. Sci.-Basel 8(9), 1567 (2018). 10.3390/app8091567

[12] Jung D. et al., “Recent progress on near-infrared photoacoustic imaging: imaging modality and organic semiconducting agents,” Polymers 11(10), 1693 (2019). 10.3390/polym11101693 PMC6836006 31623160

[r13] Hong G. et al., “Near-infrared fluorophores for biomedical imaging,” J. Biomed. Opt. 1(1), 0010 (2017). JBOPFO 1083-3668 10.1038/s41551-016-0010

[r14] Upputuri P. K. et al., “Photoacoustic imaging in the second near-infrared window: a review,” J. Biomed. Opt. 24(4), 040901 (2019). JBOPFO 1083-3668 10.1117/1.JBO.24.4.040901 PMC6990072 30968648

[15] Nachabe R. et al., “Diagnosis of breast cancer using diffuse optical spectroscopy from 500 to 1600 nm: comparison of classification methods,” J. Biomed. Opt. 16(8), 087010 (2011). JBOPFO 1083-3668 10.1117/1.3611010 21895337

[r16] Sekar S. K. V. et al., “Diffuse optical characterization of collagen absorption from 500 to 1700 nm,” J. Biomed. Opt. 22(1), 015006 (2017). JBOPFO 1083-3668 10.1117/1.JBO.22.1.015006 28138693

[17] Wilson R. H. et al., “Review of short-wave infrared spectroscopy and imaging methods for biological tissue characterization,” J. Biomed. Opt. 20(3), 030901 (2015). JBOPFO 1083-3668 10.1117/1.JBO.20.3.030901 25803186 PMC4370890

[18] Yan Y. et al., “Photoacoustic imaging of the uterine cervix to assess collagen and water content changes in murine pregnancy,” Biomed. Opt. Express 10(9), 4643–4655 (2019). BOEICL 2156-7085 10.1364/BOE.10.004643 31565515 PMC6757472

[19] Zhu Y. et al., “Identifying intestinal fibrosis and inflammation by spectroscopic photoacoustic imaging: an animal study in vivo,” Biomed. Opt. Express 9(4), 1590–1600 (2018). BOEICL 2156-7085 10.1364/BOE.9.001590 29675304 PMC5905908

[20] Wang P. et al., “Mapping lipid and collagen by multispectral photoacoustic imaging of chemical bond vibration,” J. Biomed. Opt. 21(9), 096010 (2012). JBOPFO 1083-3668 10.1117/1.JBO.21.9.096010 PMC3442104 23085911

[21] Wang P. et al., “Bond-selective imaging of deep tissue through the optical window between 1600 and 1850 nm,” J. Biophotonics 5(1), 25–32 (2011). 10.1002/jbio.201100102 22125288 PMC3404621

[22] Allen T. J. et al., “Spectroscopic photoacoustic imaging of lipid-rich plaques in the human aorta in the 740 to 1400 nm wavelength range,” J. Biomed. Opt. 17(6), 061209 (2012). JBOPFO 1083-3668 10.1117/1.JBO.17.6.061209 22734739

[23] Wang L. V. Wu H., Biomedical Optics: Principles and Imaging, Wiley, Hoboken, New Jersey (2007).

[24] Yao D. K. et al., “Photoacoustic measurement of the Grüneisen parameter of tissue,” J. Biomed. Opt. 19(1), 017007 (2014). JBOPFO 1083-3668 10.1117/1.JBO.19.1.017007 PMC3904038 24474512

[25] Buiteveld H. et al., “Optical properties of pure water,” Proc. SPIE 2258, 174–183 (1994). PSISDG 0277-786X 10.1117/12.190060

