# Supplementary material for: Effective photoacoustic absorption spectrum for collagen-based tissue imaging
Source: J Biomed Opt. 2020 May 13;25(5):056002. doi: 10.1117/1.JBO.25.5.056002 (PMC7219632; doi:10.1117/1.JBO.25.5.056002)
Supplement: Supplementary file 1 [file JBO_025_056002_SD001.docx]

**Manuscript tile: Effective photoacoustic absorption spectrum for collagen-based tissue imaging**


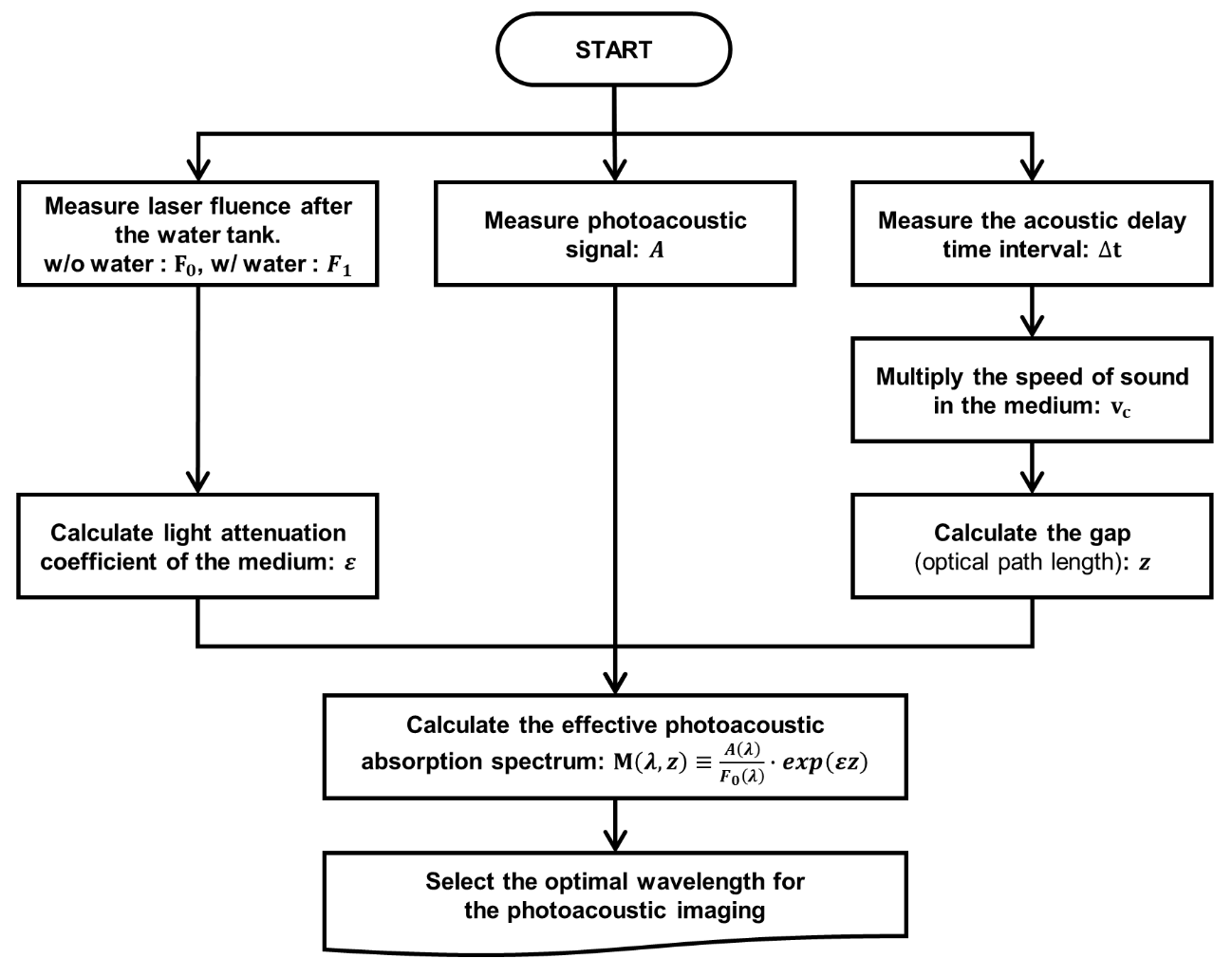


**Fig. S1** Flow chart for the effective photoacoustic absorption spectrum.


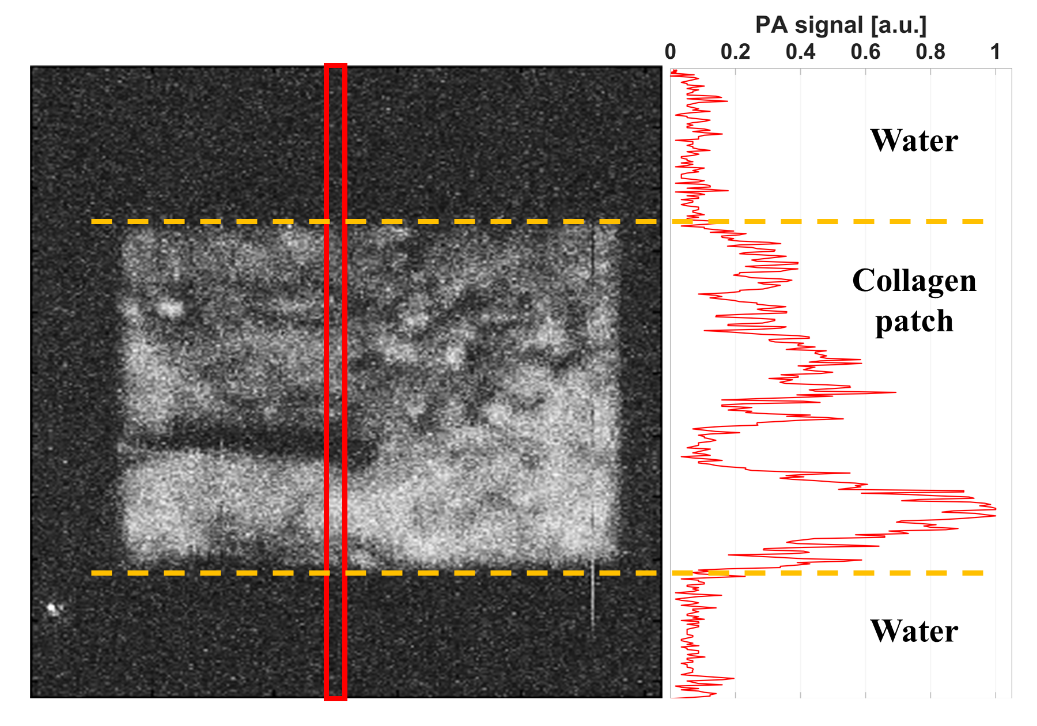


**Fig. S2** Photoacoustic MAP image of the collagen patch and corresponding averaged lateral PA signal profile (8 profiles) of the selected region on interest (ROI).


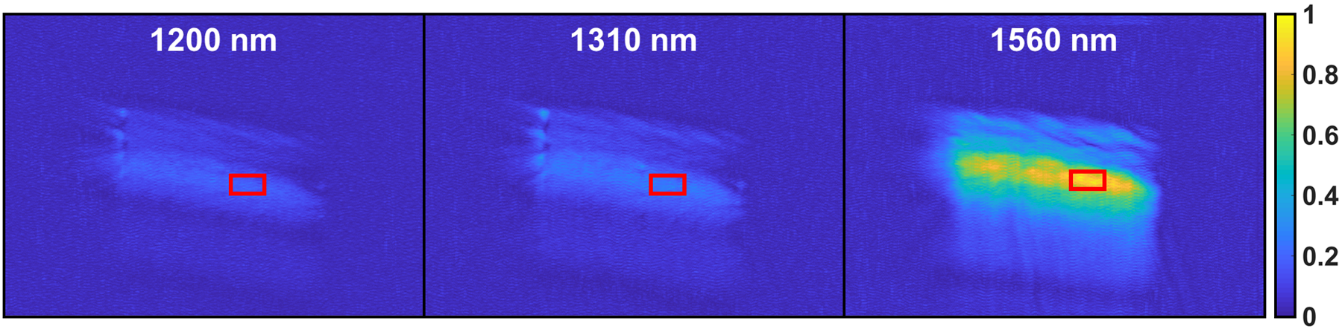


Fig. S3 B-scan PA images of the collagen patch obtained at wavelengths of 1200, 1310, and 1560 nm.
